# Exploratory Study Using Urinary Volatile Organic Compounds for the Detection of Hepatocellular Carcinoma

**DOI:** 10.3390/molecules26092447

**Published:** 2021-04-22

**Authors:** Ayman S. Bannaga, Heena Tyagi, Emma Daulton, James A. Covington, Ramesh P. Arasaradnam

**Affiliations:** 1 Department of Gastroenterology and Hepatology, University Hospital, Coventry CV2 2DX, UK; ayman.bannaga@warwick.ac.uk; 2 Warwick Medical School, University of Warwick, Coventry CV4 7HL, UK; 3 School of Engineering, University of Warwick, Coventry CV4 7AL, UK; heena.tyagi@warwick.ac.uk (H.T.); e.daulton@warwick.ac.uk (E.D.); j.a.covington@warwick.ac.uk (J.A.C.); 4 Faculty of Health & Life Sciences, Coventry University, Coventry CV1 5FB, UK; 5 Leicester Cancer Research Centre, University of Leicester, Leicester LE1 7RH, UK

**Keywords:** urinary biomarkers, hepatocellular carcinoma, diagnosis, volatile organic compounds, headspace analysis

## Abstract

Hepatocellular carcinoma (HCC) biomarkers are lacking in clinical practice. We therefore explored the pattern and composition of urinary volatile organic compounds (VOCs) in HCC patients. This was done in order to assess the feasibility of a potential non-invasive test for HCC, and to enhance our understanding of the disease. This pilot study recruited 58 participants, of whom 20 were HCC cases and 38 were non-HCC cases. The non-HCC cases included healthy individuals and patients with various stages of non-alcoholic fatty liver disease (NAFLD), including those with and without fibrosis. Urine was analysed using gas chromatography–ion mobility spectrometry (GC–IMS) and gas chromatography–time-of-flight mass spectrometry (GC–TOF-MS). GC–IMS was able to separate HCC from fibrotic cases with an area under the curve (AUC) of 0.97 (0.91–1.00), and from non-fibrotic cases with an AUC of 0.62 (0.48–0.76). For GC-TOF-MS, a subset of samples was analysed in which seven chemicals were identified and tentatively linked with HCC. These include 4-methyl-2,4-*bis*(*p*-hydroxyphenyl)pent-1-ene (2TMS derivative), 2-butanone, 2-hexanone, benzene, 1-ethyl-2-methyl-, 3-butene-1,2-diol, 1-(2-furanyl)-, bicyclo(4.1.0)heptane, 3,7,7-trimethyl-, [1S-(1a,3β,6a)]-, and sulpiride. Urinary VOC analysis using both GC–IMS and GC-TOF-MS proved to be a feasible method of identifying HCC cases, and was also able to enhance our understanding of HCC pathogenesis.

## 1. Introduction

Hepatocellular carcinoma (HCC) is the third most common cause of cancer-related death worldwide [1]. In most cases, HCC is considered a consequence of liver fibrosis/cirrhosis, with chronic viral hepatitis, alcoholic liver disease, and non-alcoholic fatty liver disease (NAFLD) being the most common underlying causes [2]. Early detection of HCC is usually reliant on ultrasound scan (USS) surveillance of cirrhotic patients. In these patients, the USS detection of HCC lesions varies according to the experience of the USS operator. Detection sensitivity can range from 40% to 80%. Another test that can be used for cirrhotic patients is the serum marker alpha-fetoprotein (AFP). AFP has poor sensitivity and relies on the cut-off being applied. Due to this, the clinical guidelines in 2018 recommended that AFP should no longer be used in routine clinical practice [3,4].

HCC diagnosis relies on advanced contrast-enhanced scans, which are either computed tomography (CT) or magnetic resonance (MR). HCC tissue biopsy is reserved for the confirmation of inconclusive HCC lesions found on a scan, or for determining the choice of palliative chemotherapy in case there is need to differentiate between HCC and other hepatobiliary malignancies [3,4,5]. HCC is often diagnosed late due to inaccessibility to CT and/or MR scans, especially in low-resource settings. Another factor involved in delayed diagnosis is the absence of symptoms until late in the disease. In addition, HCC has no approved screening programme for the general population—unlike colorectal, breast, or cervical cancers [1,2,3,4,5].

Given these factors there is still a need for ways to diagnose and understand the pathogenesis of HCC. One of the described mechanisms in HCC pathogenesis involves the impairment of hepatic metabolic pathways. The literature suggests that HCC development could be related to the malfunction of the cytochrome polysubstrate 450 (CYP450). These are heme-containing monooxygenases located in the endoplasmic reticula of the hepatic cells. The main function of cytochromes is to detoxify chemicals that could be harmful to tissues. However, this detoxification may produce harmful metabolites that could disrupt the hepatic cellular DNA division mechanisms required to maintain hepatic cellular proliferation, with subsequent cancer formation [6,7,8,9,10,11]. Because HCC is a vascularized tumour, we hypothesized that the byproducts of CYP450, including different volatile organic compounds (VOCs), would be found in the urine following the homeostatic HCC cells’ secretion of these compounds into systemic circulation, and subsequent kidney filtration. We therefore designed a pilot study with the aim of assessing this hypothesis.

## 2. Results

**Figure 1:**
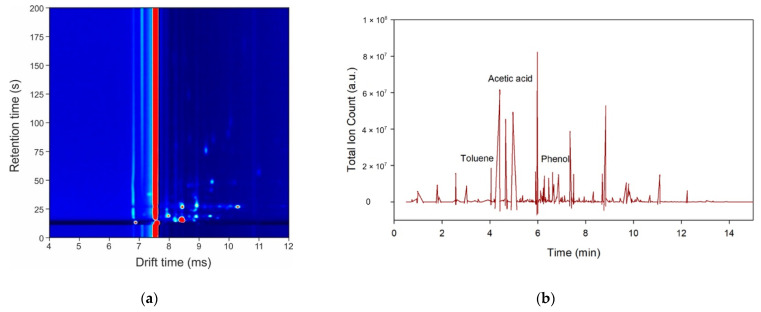
Example outputs of the instruments to a urine sample: (**a**) gas chromatography-ion mobility spectrometry (GC-IMS); (**b**) gas chromatography-time-of-flight mass spectrometry (GC–TOF-MS).

Figure 1a,b shows the outputs from GC-IMS and GC-TOF-MS, respectively. For the GC–IMS output, the background is defined in blue, with the red peaks showing areas of high intensity. The long red line is the output of the instrument to the carrier gas (in this case, nitrogen). The results show that we were able to separate different chemicals within the urine sample without saturating the machine and without chemical overlap. For the GC-TOF-MS output, we see a broad range of chemical peaks throughout the spectra, with good separation. On average, the total number of peaks detected using GC-TOF-MS, after analysing HCC and fibrosis samples, was 112, and the total number of peaks detected among HCC and non-fibrosis samples was 74. Similarly, for fibrosis and non-fibrosis samples, 79 peaks were detected on average.

### 2.1. Results from GC-IMS

**Figure 2:**
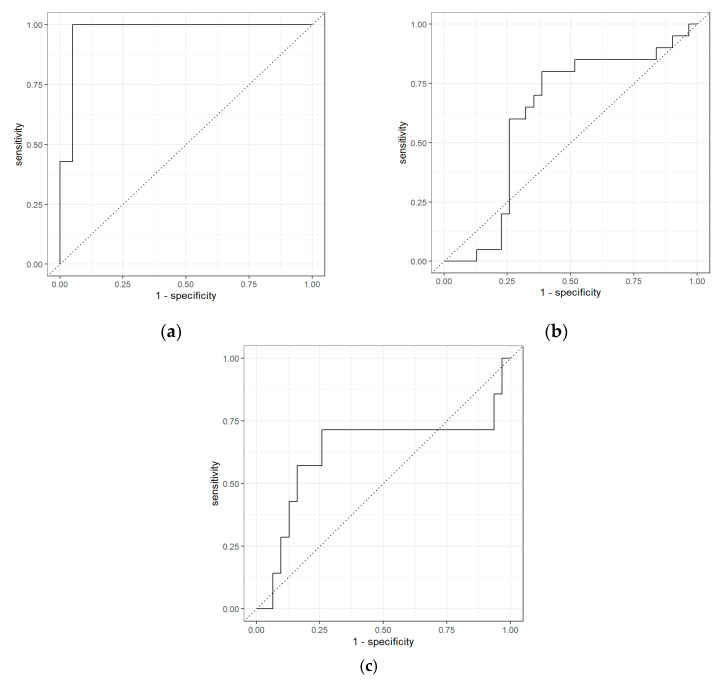
Receiver operating characteristic (ROC) curves for the GC–IMS analysis: (**a**) HCC vs. fibrosis; (**b**) HCC vs. non-fibrosis; (**c**) fibrosis vs. non-fibrosis.

Table 1 shows the results of the separation of those with HCC from non-HCC patients with liver fibrosis. The area under the curve (AUC), sensitivity, and specificity were 0.97, 0.43, and 0.95, respectively. Conversely, the separation of those with HCC compared to non-HCC patients without liver fibrosis shows modest separation, with an AUC, sensitivity, and specificity of 0.62, 0.60, and 0.74, respectively. Comparison of both fibrosis and non-fibrosis patients revealed an AUC, sensitivity, and specificity of 0.63, 0.29, and 0.90, respectively. The receiver operating characteristic (ROC) curves for the different liver groups, using GC-IMS, are presented in Figure 2. The optimal threshold values were applied for the comparison of HCC and fibrosis samples, HCC and non-fibrosis samples, and fibrosis and non-fibrosis samples, and were 0.39, 0.35, and 0.52, respectively.

The results showed that the diagnostic tests gave four false positives for comparison between HCC and fibrosis samples, eight false positive tests for HCC and non-fibrosis samples, and only three false positive tests for fibrosis and non-fibrosis samples. Moreover, the number of false negative tests for HCC and fibrosis samples was only 1, whereas the number of false negative tests for HCC and non-fibrosis samples, and for fibrosis and non-fibrosis samples, was 12 and 5, respectively.

### 2.2. Results from GC-TOF-MS Chemical Identification

Test accuracy for HCC and non-HCC cases using GC–TOF-MS is provided in Supplementary Materials Table S2 and Figure S1. This includes ROC curves for the different liver disease groups. From the total list of more than 200 chemicals identified using the National Institute of Standards and Technology (NIST) software, 5 were found to be statistically significant between the groups, with *p*-values of <0.05. Further analysis was undertaken comparing HCC with fibrosis and with non-fibrosis, and an additional two chemicals were identified from HCC versus fibrosis in the same way. No additional chemicals were identified when comparing HCC with non-fibrosis. These chemicals are listed in Table 2, with numbers 1–5 for HCC vs. non-HCC, and the remaining two associated with HCC vs. fibrosis. This table also includes the chemical retention time, the *p*-value between the groups, and whether the abundance of a chemical increased or decreased with HCC. We have not attempted to quantify these changes here due to the small sample size.

In addition, fibrosis and non-fibrosis samples were analysed in the same way. Table S3 in the Supplementary Materials provides a list of the relevant chemicals found in this analysis.

## 3. Discussion

In this study we investigated the use of VOCs as a means of providing biomarkers for the diagnosis of HCC. Here, VOCs were analysed using GC-IMS and GC-TOF-MS, which we have previously used in other clinical studies [12,13,14]. Importantly, this study further consolidates existing published studies utilizing urinary VOCs for cancer detection. The non-HCC group included both those with and without liver fibrosis, to reflect clinical HCC screening scenarios. The high specificity of 0.95 (0.86–1.00) in separating HCC from those with liver fibrosis offers important insights into the role of urinary VOCs as a screening modality. The hypothesis that the hepatic CYP450 byproducts (VOCs) related to HCC could be detected in different biological samples has been previously described. Two studies have shown that VOCs can be detected in the headspace of incubated in vitro HCC cells, supporting the use of VOC analysis for the assessment of hepatic enzyme function, as well as for the prediction of HCC progression and metastasis [15,16]. Qin et al. [17] utilized VOCs in the breath to identify HCC, independent of AFP levels or the disease’s clinical stage. A recent study by Miller-Atkins et al. [18] showed that the use of 22 VOCs in the breath could detect HCC with 0.73 sensitivity, compared with 0.53 for AFP in the same cohort.

Urine is a stable sample medium, and easier to collect for VOC analysis [19]. We have previously reported that urinary VOC analysis using solid-phase microextraction (SPME) was able to differentiate HCC and non-liver disease cases. The SPME AUC for HCC with negative alpha fetoprotein (AFP) was 0.68, and it rose to 0.83 when combined with raised AFP [20]. This was comparable to current findings reported here, where the HCC AUC was 0.62 using GC-IMS, and 0.79 using GC-TOF-MS. The study reported here also demonstrated the feasibility of urinary VOCs for differentiating between non-fibrotic, fibrotic, and HCC cases, as demonstrated in Table 1 and Supplementary Tables S1 and S2.

Using GC-TOF-MS, we tentatively identified seven VOCs related to HCC, as shown in Table 2. Though we did not perform verification and quantification of these chemicals, we did undertake a search of these VOCs in relation to the development of HCC as per the current literature. We found out that the most described VOC in HCC was 2-butanone. In experimental models, exposure to 2-butanone led to hepatotoxicity by potentiating dihydronicotinamide adenine dinucleotide phosphate (NADPH) cytochrome c reductase activity, along with the concentration of cytochrome P450 enzymes. In addition, 2-butanone exposure in these models, concomitantly with the known hepatocarcinogenic agent carbon tetrachloride (CCI4), accelerated the formation of hepatotoxic metabolites and HCC. 2-Butanone was also found to inhibit the activity of membrane-bound monoamine oxidase. This is important because monoamine oxidase was found to suppress HCC metastasis and progression by inhibiting the adrenergic system and its transactivation of epidermal growth factor receptor (EGFR) signalling [21,22,23,24,25,26,27,28,29,30]. In human studies, 2-butanone was found in the breath of HCC patients, and was found to have the best diagnostic value among other organic compounds [17]. In NAFLD paediatric patients, 2-butanone appeared at significantly higher levels in the faeces and was related to faecal *Lachnospiraceae*—a family of anaerobic, spore-forming bacteria. Additionally, the study found that *Oscillospirae* decrease relative to 2-butanone upregulation [31]. 2-Butanone was found to be elevated in cirrhotic patients who underwent liver transplantation [32]. 2-Butanone levels in the blood were found to be significantly discriminant in liver cancer patients, in comparison to healthy individuals [33]. In breath studies looking into cirrhotic and non-cirrhotic liver patients, serum bilirubin showed a positive correlation with 2-butanone. The 2-butanone in the breath also distinguished different classes of liver cirrhosis, demonstrated by Child-Turcotte-Pugh (CTP) scores of A, B and C [34,35].

We also tentatively identified 4-methyl-2,4-*bis*(*p*-hydroxyphenyl)pent-1-ene (MBP), which is a derivative of bisphenol A (BPA), a major pollutant. In the liver, MBP metabolic activation from BPA occurs via the cytochrome P450 system [36]. MBP can induce the function of oestrogen in experimental models via activation of the oestrogen receptor (ER) [37]. In patients with HCC, ERs are present and functional in around 50% of cases, but their role in promoting carcinogenesis is still not fully clear [38]. The presence of urinary MBP in HCC patients in this study suggests that MBP plays a role in HCC, perhaps via the activation of ERs, but this requires further research.

Another VOC possibly found in this study related to HCC is 2-hexanone, which was found to have a potentiating effect on the hepatotoxic agent chloroform, and subsequent liver injury, in experimental animal models [39,40]. The mechanism for this was found to be due to the induction of the CYP450 system [41,42,43]. Chronic inhalation of an isomer of 2-hexanone (methyl isobutyl ketone, MIBK) was found to cause hepatocellular adenomas and HCC in mice [44,45,46]. This was shown to be in part due to the activation of the pregnane X and constitutive androstane nuclear receptors; these receptors are responsible for the regulation of CYP450 activity [44].

Benzene, 1-ethyl-2-methyl- has been identified as a blood biomarker of HCC in a study using SPME-GC-MS [47]. Sulpiride is another chemical found in our study that is closely related to many chronic liver diseases. In particular, sulpiride was found to be related to biliary liver cirrhosis [48], NAFLD [49], and cholestatic hepatitis [50]. Though it has not been identified as a biomarker for HCC, the presence of sulpiride indicates that it may be a significant chemical for HCC. A study has suggested 3-butene-1,2-diol, 1-(2-furanyl)- as an important VOC for lung cancer [51], but it has not been verified as an HCC biomarker. Similarly, bicyclo[4.1.0]heptane, 3,7,7-trimethyl-, [1S-(1a,3β,6a)]-, found in our study, has not been identified as a biomarker. Further investigation is needed to confirm these chemicals in a larger cohort.

Our study was limited in not accounting for other factors that can be involved in the production of VOCs, such as occupational environmental factors, diet, smoking, and drug use. Another limitation was the small number of study participants. Nevertheless, this study has answered the question of whether VOCs related to the function of CYP450 in HCC can be detected in the urine. In particular, as discussed earlier, the tentative identification of urinary VOCs in this study has been seen previously in various experimental and clinical studies. The strong literature around 2-butanone encourages further study to identify the exact biochemical pathways of this compound during HCC pathogenesis. However, we did not validate these chemicals, nor did we quantify them; this effort will be undertaken in a larger study. In addition, the data from the GC-IMS system were analysed using a pattern recognition approach, and we did not attempt to identify chemical components. Again, we propose to look further into this in the next study.

## 4. Materials and Methods

This pilot study was approved by the Coventry and Warwickshire and Northeast Yorkshire NHS Ethics Committees (Ref 18717 and Ref 260179). The study conformed to the ethical principles of the Declaration of Helsinki. Study participants were recruited from University Hospital Coventry and the Warwickshire NHS Trust, UK. All participants provided written informed consent. Five-millilitre urine samples were collected into universal bottles from each study participant. These samples were then immediately frozen at −80 °C within 1 to 2 h. The samples were then stored until further sample analysis at the end of the recruitment process. We have previously tested the stability of urine samples in storage, and all methods were in line with these findings [52,53].

### 4.1. Study Characteristics

There were a total of 58 participants. These included 20 HCC cases and 38 non-HCC cases. The non-HCC cases were recruited from two sources in order to decrease bias: The first source consisted of healthy individuals without liver disease. The second source consisted of patients with different stages of NAFLD. The advantage here is that these patients represent those at risk of becoming HCC cases in the future. The non-HCC cases were then further divided into 31 non-fibrotic and 7 fibrotic/cirrhotic cases. The exclusion criteria were pregnancy and age <18 years. All of the participants were recruited prior to any anticancer treatment.

HCC diagnosis was made according to the current international guidelines, with all inconclusive cases being confirmed by a liver biopsy. Liver fibrosis/cirrhosis was confirmed by clinical examination and different radiological tests. In case of ambiguity about the clinical diagnosis, a liver biopsy was performed so as to ascertain the cause of the liver disease, and to look for the presence or absence of liver fibrosis/cirrhosis. We further collected other clinical covariates of interest, including gender, age at the time of urine sampling, history of absence or presence of diabetes, and the extent of HCC spread. We also collected liver function tests at the time of urine sampling, including AFP, alanine aminotransferase (ALT), alkaline phosphatase (ALP), albumin, and bilirubin. The study participants’ characteristics are further detailed in Table 3.

### 4.2. GC-IMS Methodology

Samples were shipped from University Hospital Coventry and from Warwickshire in universal sample containers, on dry ice, to the School of Engineering, University of Warwick, where they were stored at −20 °C until use. Prior to testing, the samples were thawed overnight in a laboratory fridge at 4 °C. Once thawed, 5 mL of each urine sample was aliquoted into 20 mL glass vials (Thames Restek, UK), and sealed with a PTFE crimp cap (Thames Restek, UK). Samples were then analysed using a FlavourSpec GC-IMS (G.A.S, Dortmund, Germany). The FlavourSpec was fitted with a CombiPAL autosampler, allowing for high-throughput automatic analysis of the samples. The samples were loaded into a cooled autosampler tray, keeping the samples at 4 °C. Each sample was heated to 40 °C and then agitated for 10 min prior to analysis. A 0.5 mL sample of the headspace was then taken using the autosampler syringe and injected directly into the GC-IMS for sampling. The GC–IMS settings were as follows: drift gas flow of 150 mL/m, and a carrier gas flow rate of 20 mL/min. The drift gas used was 99.99% nitrogen. The IMS was heated to 45 °C (T1), the GC to 40 °C (T2), the injector to 80 °C (T3), the T4 transfer line to 80 °C, and the T5 transfer line to 45 °C. Sample analysis took 10 min. Once completed, the data acquired were viewed using LAV software (G.A.S, Dortmund, Germany) and then exported for further analysis. This method has been developed over several urinary VOC studies, and is designed to maximize information content and chemical separation [12,54]. This includes the volume of urine, agitation period, and temperature. For quality control, blank samples were added at the beginning and end of each run, with the instrument having regular calibration checks run. Furthermore, the information content of each sample was checked, which included a visual inspection of each sample file.

### 4.3. GC-TOF-MS Methodology

A subset of samples was also analysed using GC-TOF-MS (Markes International, UK), with a UNITY-xr thermal desorber and ULTRA-xr autosampler (Markes International, UK). Urine samples for GC-TOF-MS were aliquoted as outlined, with about 5 mL of each sample in a 20 mL vial, which was sealed with a crimp camp. The headspace of each urine sample was then adsorbed onto a Markes bio-monitoring tube (C2-AAXX-5149). The septum of the vial was pierced, and the sorbent tube pushed through into the headspace in the vial. The samples were then heated to 40 °C for 20 min, before a pump was attached to the sorbent tube and the sample was pulled through onto the sorbent bed of the tube for 20 min whilst still being heated to 40 °C. Once complete, the tube was removed from the vial and placed into the Markes ULTRA-xr autosampler. The ULTRA-xr autosampler was set to run with a standby split of 150 °C, and a GC temperature ramp of 20 °C per minute, heating from 40 °C to 280 °C with a GC run time of 25 min. The samples were each pre-purged for 1 min, following which the sorbent tube was desorbed onto the trap for 10 min at 250 °C. Once complete, the trap was purged for a further minute and then cooled to 30 °C, before being heated to 300 °C for 3 min. Post-analysis, a dynamic baseline correction (DBS) was applied using the native TOF-DS software, and the chromatogram was integrated and deconvoluted with the following settings: global height reject of 10,000, global width reject of 0.01, baseline threshold of 3, and global area reject of 10,000. The peaks identified were then compared with the NIST list, with a match (forward and reverse) factor of 450, to identify the compounds present. As with GC–IMS, this method has been used in a number of VOC studies, including those associated with cancer, and has been previously reported on [52].

### 4.4. Statistical Analysis

The analysis of the data was undertaken using our previously reported data analysis pipeline for GC-IMS and GC-TOF-MS data, using “R” (version 3.6.3) [12,13,14]. In brief, for GC–IMS data, we applied a two-stage pre-processing step. This was undertaken because the dataset has high dimensionality (typically 11 million data points), but low chemical information. The first step was to crop the central section of the output data, where all of the chemical information is located. This was followed by the application of a threshold, below which all values were given a value of zero. This was undertaken to remove the background, leaving just the chemical information. The crop parameters were manually selected, and the same values were applied to all of the data. The threshold was defined by the value of the background noise. The data were then processed using a 10-fold cross validation. Here, the data were split into a 90% training set and a 10% test set. Within each fold, a Wilcoxon rank sum test was undertaken, and the 100 features with the lowest *p*-value were extracted. Classification models were constructed using two classifiers (eXtreme Gradient Boosting (XGBoost), and logistic regression). This process was repeated until all of the samples had been in the test group. The results were then collated, and from the resultant probabilities, statistical parameters, including sensitivity and specificity, were calculated.

For GC-TOF-MS, a similar process was undertaken. However, in this case, we used chemical identification to create features and, due to the much lower dimensionality, these were used directly by the classifier with no additional feature reduction. A further step used here was to undertake the statistical analysis of each chemical. A non-parametric t-test was undertaken in order to calculate the *p*-value of each chemical, comparing the samples in the two groups. Those chemicals found to have a *p*-value of <0.05 were considered statistically important.

## 5. Conclusions

Urinary VOCs can identify HCC cases non-invasively. The putative VOCs are likely related to CYP450 function in HCC. Our study further highlights how urine can provide a good medium for the investigation of metabolic function in HCC for further work on the cellular level.

## Figures and Tables

**Table 1:** Statistical results from the GC–IMS analysis (95% confidence intervals are in brackets). Positive predictive value (PPV); negative predictive value (NPV).

Comparison	Classifier	AUC	Sensitivity	Specificity	PPV	NPV
HCC vs. Fibrosis	Random Forest	0.97 (0.91–1.00)	0.43 (0.13–0.75)	0.95 (0.86–1.00)	0.75 (0.33–1.00)	0.83 (0.68–0.95)
HCC vs. Non-Fibrosis	Random Forest	0.62 (0.48–0.76)	0.60 (0.41–0.78)	0.74 (0.61–0.87)	0.60 (0.42–0.78)	0.74 (0.61–0.88)
Fibrosis vs. Non-Fibrosis	Linear Regression	0.63 (0.36–0.89)	0.29 (0.00–0.60)	0.90 (0.81–0.97)	0.40 (0.00–0.83)	0.85 (0.74–0.94)

**Table 2:** List of the relevant chemicals identified using GC-TOF-MS for HCC vs. non-HCC.

No.	Retention Time (min)	Chemical	*p*-value	Abundance Change
1	15.25	4-Methyl-2,4-*bis*(*p*-hydroxyphenyl)pent-1-ene, 2TMS derivative	<0.01	Lower for HCC
2	2.5998	2-Butanone	0.03637	Higher for HCC
3	4.5684	2-Hexanone	0.04309	Lower for HCC
4	6.3215	Benzene, 1-ethyl-2-methyl-	0.04183	Lower for HCC
5	12.1318	3-Butene-1,2-diol, 1-(2-furanyl)-	0.03247	Lower for HCC
6	8.2054	Bicyclo[4.1.0]heptane, 3,7,7-trimethyl-, [1S-(1a,3ß,6a)]-	0.03553	Lower for HCC
7	13.861	Sulpiride	0.04369	Lower for HCC

**Table 3:** Clinical and biochemical characteristics of the recruited study participants at the time of obtaining their urine samples.

Covariate	HCC Cases	Non-HCC Cases
No. of Patients	20	38
Age: Mean (Range)	73 (53–84)	58.08 (29–89)
Gender: Female/Male	2/18	11/27
Cause of Liver Disease	3 Alcohol 1 HBV 1 HCV 13 NASH 2 Primary/Idiopathic	1 HBV Cirrhosis 9 NAFLD 10 NASH 6 NASH Cirrhosis 12 without Liver Disease
Histological/Radiological Features of Liver Cirrhosis: Present/Absent	16/4	7/31
Diabetes: Present/Absent	11/9	7/31
AFP: Mean (Range), KU/L	1380.60 (1–9400)	-
ALT: Mean (Range), U/L	44.60 (13–149)	50.74 (5–304)
ALP: Mean (Range), U/L	150.90 (83–326)	89.76 (53–279)
Albumin: Mean (Range), g/L	39 (24–44)	43.87 (28–50)
Bilirubin: Mean (Range), µmol/L	24.30 (5–84)	7.97 (5–21)
Stage of the HCC: Hepatic/Extra-Hepatic	13/7	-

Characteristics of the HCC and non-HCC groups. HCC diagnosis was made in line with international guidelines. Liver disease was established using a combination of radiological scans, FibroScan, laboratory markers, and histology. All covariates were collected at the time of urine collection. Abbreviations: AFP, alpha-fetoprotein; ALT, alanine aminotransferase; ALP, alkaline phosphatase; HBV, hepatitis B virus; HCV, hepatitis C virus; NAFLD, non-alcoholic fatty liver disease; NASH, non-alcoholic steatohepatitis.

## Data Availability

All data are available in this manuscript and its supplementary files.

## Supplementary Materials

The following are available online: Table S1 compares HCC with non-HCC cases using GC–IMS analysis, providing AUC, sensitivity, specificity, thresholds, negative predictive value, and positive predictive value. Table S2 compares HCC with non-HCC cases using GC–TOF-MS analysis, providing AUC, sensitivity, specificity, thresholds, negative predictive value, and positive predictive value. Table S3 shows the identified chemicals for Fibrosis vs Non-Fibrosis that were statistically relevant using GC-TOF-MS. Figure S1 provides ROCs for HCC and Fibrosis samples, HCC and Non-Fibrosis and Fibrosis and Non-Fibrosis using GC-TOF-MS.

## References

[1] (2020). International Agency for Research on Cancer; Liver; World Health Organization. http://gco.iarc.fr/today/data/factsheets/cancers/11-Liver-fact-sheet.pdf.

[2] Forner A., Reig M., Bruix J. (2018). Hepatocellular carcinoma. Lancet.

[3] Galle P.R., Forner A., Llovet J.M., Mazzaferro V., Piscaglia F., Raoul J.-L., Schirmacher P., Vilgrain V. (2018). EASL Clinical Practice Guidelines: Management of hepatocellular carcinoma. J. Hepatol..

[4] Heimbach J.K., Kulik L.M., Finn R.S., Sirlin C.B., Abecassis M.M., Roberts L.R., Zhu A.X., Murad M.H., Marrero J.A. (2018). AASLD guidelines for the treatment of hepatocellular carcinoma. Hepatol..

[5] Villanueva A. (2019). Hepatocellular Carcinoma. New Engl. J. Med..

[6] Huang Q., Tan Y., Yin P., Ye G., Gao P., Lu X., Wang H., Xu G. (2013). Metabolic Characterization of Hepatocellular Carcinoma Using Nontargeted Tissue Metabolomics. Cancer Res..

[7] Thomas M., Bayha C., Vetter S., Hofmann U., Schwarz M., Zanger U.M., Braeuning A. (2015). Activating and Inhibitory Functions of WNT/β-Catenin in the Induction of Cytochromes P450 by Nuclear Receptors in HepaRG Cells. Mol. Pharmacol..

[8] Hamamoto I., Tanaka S., Maeba T., Chikaishi K., Ichikawa Y. (1989). Microsomal cytochrome P-450-linked monooxygenase systems and lipid composition of human hepatocellular carcinoma. Br. J. Cancer.

[9] Gao P., Liu Z.-Z., Yan L.-N., Dong C.-N., Ma N., Yuan M.-N., Zhou J. (2019). Cytochrome P450 family members are associated with fast-growing hepatocellular carcinoma and patient survival: An integrated analysis of gene expression profiles. Saudi J. Gastroenterol..

[10] Tsutsumi M., Matsuda Y., Takada A. (1993). Role of ethanol-inducible cytochrome P-450 2E1 in the development of hepatocellular carcinoma by the chemical carcinogen, N-nitrosodimethylamine. Hepatology.

[11] Eun H.S., Cho S.Y., Lee B.S., Seong I.O., Kim K. (2018). HProfiling cytochrome P450 family 4 gene expression in human hepatocellular carcinoma. Mol. Med. Rep..

[12] Daulton E., Wicaksono A.N., Tiele A., Kocher H.M., Debernardi S., Crnogorac-Jurcevic T., Covington J.A. (2021). Volatile organic compounds (VOCs) for the non-invasive detection of pancreatic cancer from urine. Talanta.

[13] Tiele A., Wicaksono A., Daulton E., Ifeachor E., Eyre V., Clarke S., Timings L., Pearson S., A Covington J., Li X. (2019). Breath-based non-invasive diagnosis of Alzheimer’s disease: A pilot study. J. Breath Res..

[14] Daulton E., Wicaksono A., Bechar J., Covington J.A., Hardwicke J. (2020). The Detection of Wound Infection by Ion Mobility Chemical Analysis. Biosensors.

[15] Mochalski P., Sponring A., King J., Unterkofler K., Troppmair J., Amann A. (2013). Release and uptake of volatile organic compounds by human hepatocellular carcinoma cells (HepG2) in vitro. Cancer Cell Int..

[16] Haick H., Amal H., Ding L., Liu B., Tisch U., Xu Z.-Q., Shi D.-Y., Zhao Y., Chen J., Sun R.-X. (2012). The scent fingerprint of hepatocarcinoma: In-vitro metastasis prediction with volatile organic compounds (VOCs). Int. J. Nanomed..

[17] Qin T., Liu H., Song Q., Song G., Wang H.-Z., Pan Y.-Y., Xiong F.-X., Gu K.-S., Sun G.-P., Chen Z.-D. (2010). The Screening of Volatile Markers for Hepatocellular Carcinoma. Cancer Epidemiology Biomarkers Prev..

[18] Miller-Atkins G., Acevedo-Moreno L., Grove D., Dweik R.A., Tonelli A.R., Brown J.M., Allende D.S., Aucejo F., Rotroff D.M. (2020). Breath Metabolomics Provides an Accurate and Noninvasive Approach for Screening Cirrhosis, Primary, and Secondary Liver Tumors. Hepatol. Commun..

[19] Becker R. (2020). Non-invasive cancer detection using volatile biomarkers: Is urine superior to breath?. Med. Hypotheses.

[20] Bannaga A.S.I., Kvasnik F., Persaud K.C., Arasaradnam R.P. (2020). Differentiating cancer types using a urine test for volatile organic compounds. J. Breath Res..

[21] Traiger G.J., Bruckner J.V., Jiang W., Dietz F.K., Cooke P.H. (1989). Effect of 2-butanol and 2-butanone on rat hepatic ultrastructure and drug metabolizing enzyme activity. J. Toxicol. Environ. Heal. Part A.

[22] Toftgard R., Nilsen O.G., Gustafsson J.Å. (1981). Changes in rat liver microsomal cytochrome P-450 and enzymatic activities after the inhalation of n-hexane, xylene, methyl ethyl ketone and methylchloroform for four weeks. Scand. J. Work. Environ. Heal..

[23] Wlodzimirow K., Abu-Hanna A., Schultz M., Maas M., Bos L., Sterk P., Knobel H., Soers R., Chamuleau R.A. (2014). Exhaled breath analysis with electronic nose technology for detection of acute liver failure in rats. Biosens. Bioelectron..

[24] Raunio H., Liira J., Elovaara E., Riihimäki V., Pelkonen O. (1990). Cytochrome P450 isozyme induction by methyl ethyl ketone and m-xylene in rat liver. Toxicol. Appl. Pharmacol..

[25] Peng H., Raner G., Vaz A., Coon M. (1995). Oxidative Cleavage of Esters and Amides to Carbonyl Products by Cytochrome P450. Arch. Biochem. Biophys..

[26] Brown E.M., Hewitt W.R. (1984). Dose-response relationships in ketone-induced potentiation of chloroform hepato- and nephrotoxicity. Toxicol. Appl. Pharmacol..

[27] Raymond P., Plaa G.L. (1995). Ketone potentiation of haloalkane-induced hepato- and nephrotoxicity. I. dose-response relationships. J. Toxicol. Environ. Heal. Part A.

[28] Fowler C.J., Oreland L. (1980). The effect of lipid-depletion on the kinetic properties of rat liver monoamine oxidase-B. J. Pharm. Pharmacol..

[29] Kinemuchi H., Sunami Y., Sudo M., Suh Y.H., Arai Y., Kamijo K. (1985). Membrane lipid environment of carp brain and liver mitochondrial monoamine oxidase. Comp. Biochem. Physiol. Part C Comp. Pharmacol..

[30] Li J., Yang X.-M., Wang Y.-H., Feng M.-X., Liu X.-J., Zhang Y.-L., Huang S., Wu Z., Xue F., Qin W.-X. (2014). Monoamine oxidase A suppresses hepatocellular carcinoma metastasis by inhibiting the adrenergic system and its transactivation of EGFR signaling. J. Hepatol..

[31] Del Chierico F., Nobili V., Vernocchi P., Russo A., De Stefanis C., Gnani D., Furlanello C., Zandonà A., Paci P., Capuani G. (2017). Gut microbiota profiling of pediatric nonalcoholic fatty liver disease and obese patients unveiled by an integrated meta-omics-based approach. Hepatology.

[32] Del Río R.F., O’Hara M., Holt A., Pemberton P., Shah T., Whitehouse T., Mayhew C. (2015). Volatile Biomarkers in Breath Associated With Liver Cirrhosis—Comparisons of Pre- and Post-liver Transplant Breath Samples. EBioMedicine.

[33] Wu S., Cai C., Cheng J., Cheng M., Zhou H., Deng J. (2016). Polydopamine/dialdehyde starch/chitosan composite coating for in-tube solid-phase microextraction and in-situ derivation to analysis of two liver cancer biomarkers in human blood. Anal. Chim. Acta.

[34] Morisco F., Aprea E., Lembo V., Fogliano V., Vitaglione P., Mazzone G., Cappellin L., Gasperi F., Masone S., De Palma G.D. (2013). Rapid “Breath-Print” of Liver Cirrhosis by Proton Transfer Reaction Time-of-Flight Mass Spectrometry. A Pilot Study. PLoS ONE.

[35] Velde S.V.D., Nevens F., Van Hee P., Van Steenberghe D., Quirynen M. (2008). GC–MS analysis of breath odor compounds in liver patients. J. Chromatogr. B.

[36] Liu S.-H., Su C.-C., Lee K.-I., Chen Y.-W. (2016). Effects of Bisphenol A Metabolite 4-Methyl-2,4-bis(4-hydroxyphenyl)pent-1-ene on Lung Function and Type 2 Pulmonary Alveolar Epithelial Cell Growth. Sci. Rep..

[37] Hirao-Suzuki M., Takeda S., Okuda K., Takiguchi M., Yoshihara S. (2018). Repeated Exposure to 4-Methyl-2,4-*bis*(4-hydroxyphenyl)pent-1-ene (MBP), an Active Metabolite of Bisphenol A, Aggressively Stimulates Breast Cancer Cell Growth in an Estrogen Receptor β (ERβ)–Dependent Manner. Mol. Pharmacol..

[38] Villa E. (2008). Role of Estrogen in Liver Cancer. Women’s Heal..

[39] Cowlen M.S., Hewitt W.R., Schroeder F. (1984). 2-Hexanone potentiation of [14C]chloroform hepatotoxicity: Covalent interaction of a reactive intermediate with rat liver phospholipid. Toxicol. Appl. Pharmacol..

[40] Hewitt L.A., Valiquette C., Plaa G.L. (1987). The role of biotransformation–detoxication in acetone-, 2-butanone-, and 2-hexanone-potentiated chloroform-induced hepatotoxicity. Can. J. Physiol. Pharmacol..

[41] Nakajima T., Elovaara E., Park S.S., Gelboin H.V., Vainio H. (1991). Immunochemical detection of cytochrome P450 isozymes induced in rat liver byn-hexane, 2-hexanone and acetonyl acetone. Arch. Toxicol..

[42] Nakajima T., Elovaara E., Okino T., Gelboin H., Klockars M., Riihimaki V., Aoyama T., Vainio H. (1995). Different Contributions of Cytochrome P450 2E1 and P450 2B1/2 to Chloroform Hepatotoxicity in Rat. Toxicol. Appl. Pharmacol..

[43] Cowlen M.S., Hewitt W.R., Schroeder F. (1984). Mechanisms in 2-hexanone potentiation of chloroform hepatotoxicity. Toxicol. Lett..

[44] Hughes B., Thomas J., Lynch A., Borghoff S., Green S., Mensing T., Sarang S., LeBaron M. (2016). Methyl isobutyl ketone-induced hepatocellular carcinogenesis in B6C3F1 mice: A constitutive androstane receptor (CAR)-mediated mode of action. Regul. Toxicol. Pharmacol..

[45] Stout M.D., Herbert R.A., Kissling G.E., Suarez F., Roycroft J.H., Chhabra R.S., Bucher J.R. (2008). Toxicity and carcinogenicity of methyl isobutyl ketone in F344N rats and B6C3F1 mice following 2-year inhalation exposure. Toxicology.

[46] National Toxicology Program (2007). Toxicology and carcinogenesis studies of methyl isobutyl ketone (Cas No. 108-10-1) in F344/N rats and B6C3F1 mice (inhalation studies). Natl. Toxicol. Prog. Tech. Rep. Ser..

[47] Xue R., Dong L., Zhang S., Deng C., Liu T., Wang J., Shen X. (2008). Investigation of volatile biomarkers in liver cancer blood using solid-phase microextraction and gas chromatography/mass spectrometry. Rapid Commun. Mass Spectrom..

[48] Ohmoto K., Yamamoto S., Hirokawa M. (1999). Symptomatic Primary Biliary Cirrhosis Triggered by Administration of Sulpiride. Am. J. Gastroenterol..

[49] Zhou X., Ren L., Yu Z., Huang X., Li Y., Wang C. (2018). The antipsychotics sulpiride induces fatty liver in rats via phosphorylation of insulin receptor substrate-1 at Serine 307-mediated adipose tissue insulin resistance. Toxicol. Appl. Pharmacol..

[50] Gustafsson F., Foster A.J., Sarda S., Bridgland-Taylor M.H., Kenna J.G. (2013). A Correlation Between the In Vitro Drug Toxicity of Drugs to Cell Lines That Express Human P450s and Their Propensity to Cause Liver Injury in Humans. Toxicol. Sci..

[51] Chen K.-C., Tsai S.-W., Zhang X., Zeng C., Yang H.-Y. (2021). The Investigation of the Volatile Metabolites of Lung Cancer from the Microenvironment of Malignant Pleural Effusion. https://www.researchsquare.com/article/rs-144572/v1.

[52] Esfahani S., Sagar N.M., Kyrou I., Mozdiak E., O’Connell N., Nwokolo C., Bardhan K.D., Arasaradnam R.P., Covington J.A. (2016). Variation in Gas and Volatile Compound Emissions from Human Urine as It Ages, Measured by an Electronic Nose. Biosensors.

[53] McFarlane M., Mozdiak E., Daulton E., Arasaradnam R., Covington J., Nwokolo C. (2020). Pre-analytical and analytical variables that influence urinary volatile organic compound measurements. PLoS ONE.

[54] Mozdiak E., Wicaksono A.N., Covington J.A., Arasaradnam R.P. (2019). Colorectal cancer and adenoma screening using urinary volatile organic compound (VOC) detection: Early results from a single-centre bowel screening population (UK BCSP). Tech. Coloproctology.

