# Supplementary material for: Exploratory Study Using Urinary Volatile Organic Compounds for the Detection of Hepatocellular Carcinoma
Source: Molecules. 2021 Apr 22;26(9):2447. doi: 10.3390/molecules26092447 (PMC8122735; doi:10.3390/molecules26092447)
Supplement: Supplementary file 1 [file molecules-26-02447-s001.zip › molecules-1177634-supplementary.pdf]

Supplementary Materials

Table S1. GC-IMS result for different liver disease groups (95% confidence intervals are in brackets). The table compares HCC with non-HCC using GC-IMS analysis, providing thresholds, AUC, sensitivity, specificity, negative predictive value and positive predictive value.

Comparisons	Classifiers	Threshold	AUC	Sensitivity	Specificity	PPV	NPV
HCC vs Fibrosis	RF	0.39	0.97 (0.91 – 1)	0.43 (0.13 – 75)	0.95 (0.86- 1)	0.75 (0.53 – 1)	0.83 (0.68 -0.96)
HCC vs Fibrosis	LR	0.39	0.92 (0.84 – 1)	0.71 (0.40 – 1)	0.9 (0.77 – 1)	0.71 (0.4 – 1)	0.9 (0.78 – 1)
HCC vs Non-Fibrosis	RF	0.35	0.62 (0.48 – 0.76)	0.6 (0.41 – 0.78)	0.74 (0.61 – 0.87)	0.6 (0.42 – 0.78)	0.74 (0.61 – 0.88)
HCC vs Non-Fibrosis	LR	0.34	0.62 (0.48 – 0.75)	0.71 (0.4 –0.68)	0.9 (0.77 – 0.78)	0.48 (0.29 – 0.65)	0.67 (0.52 – 0.8)
Fibrosis vs Non-Fibrosis	RF	0.41	0.5 (0.25 – 0.74)	0.14 (0 – 0.4)	0.97 (0.91 – 1)	0.5 (0.5 – 1)	0.83 (0.73 – 0.92)
Fibrosis vs Non-Fibrosis	LR	0.46	0.63 (0.36 – 0.89)	0.29 (0 – 0.6)	0.90 (0.81 – 0.97)	0.4 (0 – 0.83)	0.85 (0.74 – 0.94)

Table S2. GC-TOF-MS result for different liver disease groups (95% confidence intervals are in brackets). The table compares HCC with non-HCC using GC-TOF-MS analysis, providing thresholds, AUC, sensitivity, specificity, negative predictive value and positive predictive value.

Comparisons	Classifiers	Threshold	AUC	Sensitivity	Specificity	PPV	NPV
HCC vs Fibrosis	LR	0.32	0.79 (0.50 – 1)	0.71 (0.40 – 1)	0.66 (0.33 – 1)	0.66 (0.23 – 1)	0.71 (0.40 -1)
HCC vs Fibrosis	RF	0.33	0.71 (0.40 – 0.91)	0.71 (0.40 – 1)	0.5 (0.17 – 0.86)	0.60 (0.25 – 1)	0.62 (0.40 – 0.89)
HCC vs Non-Fibrosis	LR	0.32	0.83 (0.62 – 1)	0.66 (0.33 – 1)	0.71 (0.43 – 1)	0.71 (0.40 – 1)	0.67 (0.33 – 1)
HCC vs Non-Fibrosis	RF	0.31	0.71 (0.43 – 0.95)	0.66 (0.30 – 1)	0.43 (0.14 – 0.75)	0.60 (0.20 – 1)	0.50 (0.20 – 0.80)
Fibrosis vs Non-Fibrosis	LR	0.34	0.84 (0.58 – 1)	0.86 (0.60 – 1)	0.57 (0.25 – 0.88)	0.80 (1 – 0.5)	0.67 (0.38 – 0.90)
Fibrosis vs Non-Fibrosis	RF	0.47	0.60 (0.33 – 0.88)	0.71 (0.40 – 1)	0.57 (0.25 – 0.87)	0.66 (0.33 – 1)	0.62 (0.22 – 0.88)

Table S3. GC-TOF-MS identified chemicals that were statistically relevant for fibrosis vs non-fibrosis.

No.	Retention time (min)	Chemical	*p*-value
1	12.13	3-Butene-1,2-diol, 1-(2-furanyl)-	<0.01
2	12.26	Isopropyl myristate	0.00361
3	5.59	Octane, 2,2,6-trimethyl-	0.00457
4	6.27	Benzene, 1-ethyl-2-methyl-	0.04183
5	9.83	Biphenyl	0.04714

Figure S1 provides the ROC curves for the analysis undertaken by GC-TOF-MS (a) HCC and Fibrosis samples, (b) HCC and Non-Fibrosis and (c) Fibrosis and Non-Fibrosis

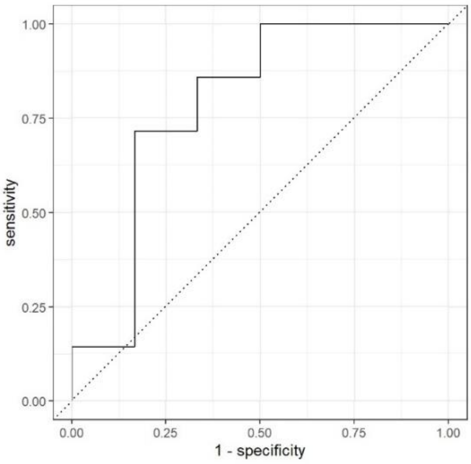

(a)

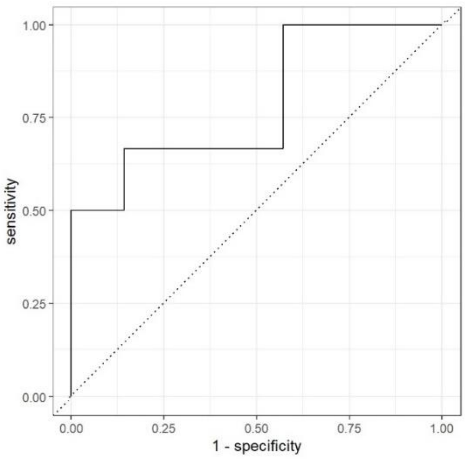

(b)

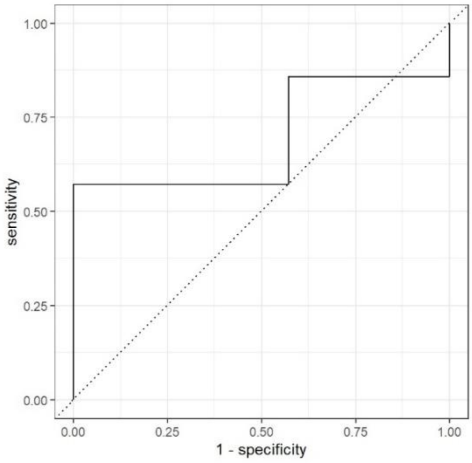

(c)
